# Comparing antibiotic treatment for leptospirosis using network meta-analysis: a tutorial

**DOI:** 10.1186/s12879-016-2145-3

**Published:** 2017-01-05

**Authors:** Cho Naing, Simon A. Reid, Kyan Aung

**Affiliations:** 1 School of Postgraduate Studies, International Medical University, Kuala Lumpur, 5700 Malaysia; 2 School of Public Health, University of Queensland, Brisbane, Australia; 3 School of Medicine, International Medical University, Kuala Lumpur, Malaysia

## Abstract

**Background:**

Network meta-analysis consists of simultaneous analysis of both direct comparisons of interventions within randomized controlled trials and indirect comparisons across trials based on a common comparator. In this paper, we aimed to characterise the conceptual understanding and the rationale for the use of network meta-analysis in assessing drug efficacy.

**Methods:**

We selected randomized controlled trials, assessing efficacy of antibiotics for the treatment of leptospirosis as a case study. A pairwise meta-analysis was conducted using a random effect model, assuming that different studies assessed different but related treatment effects. The analysis was then extended to a network meta-analysis, which consists of direct and indirect evidence in a network of antibiotics trials, using a suite of multivariate meta-analysis routines of STATA (*mvmeta* command). We also assessed an assumption of ‘consistency’ that estimates of treatment effects from direct and indirect evidence are in agreement.

**Results:**

Seven randomised controlled trials were identified for this analysis. These RCTs assessed the efficacy of antibiotics such as penicillin, doxycycline and cephalosporin for the treatment of human leptospirosis. These studies made comparisons between antibiotics (i.e. an antibiotic versus alternative antibiotic) in the primary study and a placebo, except for cephalosporin. These studies were sufficient to allow the creation of a network for the network meta-analysis; a closed loop in which three comparator antibiotics were connected to each other through a polygon. The comparison of penicillin versus the placebo has the largest contribution to the entire network (31.8%). The assessment of rank probabilities indicated that penicillin presented the greatest likelihood of improving efficacy among the evaluated antibiotics for treating leptospirosis.

**Conclusions:**

Findings suggest that network meta-analysis, a meta-analysis comparing multiple treatments, is feasible and should be considered as better precision of effect estimates for decisions when several antibiotic options are available for the treatment of leptospirosis.

**Electronic supplementary material:**

The online version of this article (doi:10.1186/s12879-016-2145-3) contains supplementary material, which is available to authorized users.

## Background

Systematic reviews use explicit, pre-specified methods to identify, appraise and synthesize all available evidence related to a (clinical) question of research interest. If appropriate, systematic reviews may include a quantitative data synthesis (i.e. meta-analysis), which is the statistical combination of results from ≥ 2 individual studies [1]. However, systematic reviews conventionally compare only 2 interventions, despite having the existence of more than two interventions for a disease of interest. For instance, a randomised controlled trial (RCT) on antibiotics for treating leptospirosis included three arms [2]. As such, a conventional pairwise meta-analysis may be conducted, but the comparative effectiveness of all available interventions for a given condition will not be addressed [3]. Individual pair-wise comparisons, which in isolation fall short of informing clinical decisions when there are a greater number of treatment options available [4]. A network meta-analysis (NMA), also known as mixed treatment comparison or multiple treatment comparison, is a method for simultaneous comparison of multiple treatments in a single meta-analysis [3]. It expands the scope of a traditional (conventional) pairwise meta-analysis by analysing simultaneously both direct comparisons of interventions within RCTs and indirect comparisons across trials based on a common comparator [5–7]. The multivariate approach, therefore, allows one to ‘borrow strength’ across correlated outcomes, to potentially reduce the impact of outcome reporting bias [8].

Leptospirosis is a zoonosis caused by infection with pathogenic *Leptospira* species that has a global distribution with a significant health impact, particularly in resource-poor tropical countries [9]. The clinical course in humans ranges from mild to lethal with a broad spectrum of symptoms and clinical signs [10]. A recent systematic review estimated that there are 1.03 (95% CI 0.43–1.75) million cases of leptospirosis worldwide each year and 58,900 deaths (95% CI 23,800–95,900) [11, 12], which corresponds to an estimated 2.9 million disability-adjusted life years per annum, including 2.8 million years of life lost due to premature death [9]. Thus far, the optimal treatment of leptospirosis remains a subject of debate, mainly due to the wide and biphasic clinical spectrum of the disease and the distinct pathogenesis in these two phases [13, 14].

Taken together, the objective of this study was to characterise the conceptual understanding and the rational for the use of NMA in assessing drug efficacy. As such, we used results from RCTs of antibiotics for the treatment of leptospirosis as a case study.

## Methods

### Searching studies

First, we searched for RCTs evaluating the efficacy of antibiotics for the treatment of patients with leptospirosis in electronic databases (Medline, EMBASE) up to June 2016. We used a search strategy with terms relevant to leptospirosis, RCTs and antibiotics individually and in combination (Additional file 1: Table S1). We also searched the relevant studies in the Cochrane Central Register of Controlled Trials (CENTRAL) and EBSCO CINAHL. Two investigators within the reviewing team independently screened the title and abstract retrieved from the searches. Individual studies were selected based on the following predetermined criteria in PICOS, described elsewhere [1, 15]: Population (P): those patients diagnosed with leptospirosis; Interventions (I): antibiotics; Comparisons (C): an antibiotic versus alternative antibiotic or placebo; Outcomes (O): mortality; and Study design (S): RCTs. We defined mortality as death of a patient at any follow-up time point given in the primary study, after administration of a selected treatment option. This primary outcome was chosen because it is the most important estimate of treatment efficacy.

For each identified study that met the selection criteria we extracted data on study design, study population characteristics and interventions (type of antibiotics, dosage, route of administration, day of treatment initiation and follow-up duration). We rated the methodological quality of each included RCT, using a risk of bias (RoB) tool recommended by the Cochrane Collaboration for assessment criteria. The RoB tool is a domain-based assessment to detect random sequence generation, allocation concealment, blinding in the studies (patients, assessors and physicians), incomplete outcome data, selective outcome reporting, and evidence of major baseline imbalance [15].

### Assessing the feasibility of a network meta-analysis

We assessed whether an NMA would provide a method to indirectly compare an antibiotic in terms of the specified outcomes for patients diagnosed with leptospirosis. The placebo-controlled clinical trial has a long history of being the standard for clinical investigations of new drugs [16]. Published RCTs that assessed the efficacy of antibiotics for the treatment of human leptospirosis included penicillin, doxycycline and cephalosporin. These studies made comparisons between antibiotics (i.e. an antibiotic vs alternative antibiotic) in the primary study and a placebo, except for cephalosporin. This exception might be due to the ethical issues associated with withholding treatment for a fatal illness or possibly due to the lack of sponsorship by industry. In the absence of trials involving a direct comparison of interventions, an indirect comparison can provide valuable evidence for the relative treatment effects between competing interventions [17, 18]. If we want to make best use of the evidence, it is necessary to analyse all the evidence jointly [19]. In order to do a network plot, we used the STATA command (*network map*) [20, 21].

### Statistical analysis

The number of deaths and corresponding total number of participants in each treatment arm were extracted from the included studies and used to calculate the outcome measure of treatment efficacy as an odds ratio (OR) and corresponding 95% confidence interval (CI). A pairwise meta-analysis was conducted by synthesising studies that compared the same interventions with a random effect model, assuming that different studies assessed different but related treatment effects. Between-study heterogeneity was assessed with *I* ^2^ statistics (*I* ^2^ > 50% was considered to show substantial heterogeneity) [15]. The analysis was then extended to an NMA, which consists of direct and indirect evidence in a network of antibiotics trials, using a suite of multivariate meta-analysis routines of STATA (*mvmeta* command) to evaluate the assumptions in the studies and provide graphical presentation of results [20, 21]. In the suite, the assumption of ‘consistency’ and ‘inconsistency’ in NMA was assessed using a data augmentation approach [20]. The assumption of ‘consistency’ implies that estimates of treatment effects from direct and indirect evidence are in agreement [19], where as evidence ‘inconsistency’ is the discrepancy between direct and indirect comparisons [17]. Our null hypothesis was that there was consistency between the direct and indirect evidence [19] and we would reject the null hypothesis if there was a statistically significant difference between the direct and indirect evidence comparison (*p* < 0.05).

The comparative efficacy of four antibiotics included in this review was assessed using penicillin as the reference treatment because it is the first choice antibiotic for treating leptospirosis. The probability that each antibiotic is the best among the given treatments was determine by evaluating the rank probabilities and surface under the cumulative ranking curve (SUCRA) for the efficacy results of the NMA [20, 21]. A higher probability of achieving rank 1 indicates a higher probability that treated patients will experience a greater improvement in terms of mortality outcome (i.e. more likely to survive).

The heterogeneity of the indirect comparison was assessed using *tau* ^*2*^, which examines heterogeneity because of study and study drug interaction (smaller values indicate a better model). For each outcome, one common heterogeneity parameter, tau^2^, which is the estimated standard deviation of underlying effects across studies [15] was assumed across comparisons, which corresponded to the variance of the underlying distribution. A *tau* ^*2*^ value ≥ 1 is considered to indicate relatively high intra-study variability [17]. All analysis were conducted using Stata I/C version 14.0 (Stata Corp, Txt).

## Results

### Feasibility of a network meta-analysis

**Fig. 1:**
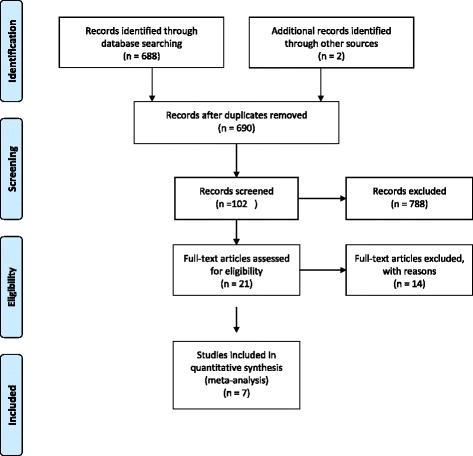
Flow Diagram of the study selection process

**Fig. 2:**
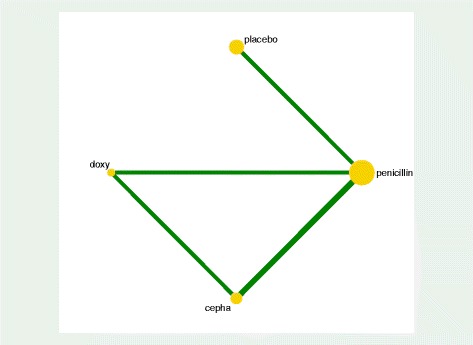
Network geometry of the antibiotics used in treating leptospirosis

Figure 1 shows the study selection process for the systematic review of antibiotic treatment of leptospirosis. We found four RCTs compared penicillin to a placebo [22–25], two RCTs comparing penicillin to a cephalosporin [2, 26], one RCT comparing doxycycline and a placebo [27], and further RCT comparing penicillin to doxycycline [2]. These seven RCTs [2, 22–27] were sufficient to allow the creation of a network for the NMA. Figure 2 shows a closed loop in which three comparator antibiotics were connected to each other through a polygon. Treatments penicillin, doxycycline, and cephalosporin (ceftriaxone or cefotaxime) were compared against each other in these trials and thus each comparison in the closed loop is informed by both direct and indirect evidence in the present leptospirosis network.

The network map shows all the available comparisons in the network using weighted nodes and the RoB level (for blinding in this case) for each comparison using colored edges. Each line joining two treatments represents a direct head-to-head comparison, providing efficacy in terms of mortality outcome. The size of the nodes is proportional to the number of studies evaluating each intervention and the thickness of the edges is proportional to the precision of each direct comparison.

### Systematic review results

The characteristics of the seven trials included in this analysis are presented in Additional file 2. RCTs identified for the current network were generally single centre, open label trials evaluating the efficacy of antibiotics in treating patients diagnosed with leptospirosis. With regard to the methodological quality of RCTs in this analysis, only one trial [26] had low RoB with regards the adequacy of blinding (Table 1).

**Table 1:** Risk of bias assessment

Study, year [ref]	Random sequences generation	Allocation concealment	Blinding of outcome assessment
Suputtamongkol, 2004 [2]	low risk	low risk	high risk
Edwards,1988 [22]	low risk	unclear	unclear
Watt, 1988 [23]	high risk	high risk	high risk
Dahler, 2000 [24]	high risk	high risk	high risk
Costa,2003 [25]	unclear	unclear	unclear risk
Panaphut, 2003 [26]	low risk	low risk	low risk
McClain, 1984 [27]	low risk	low risk	unclear

**Fig. 3:**
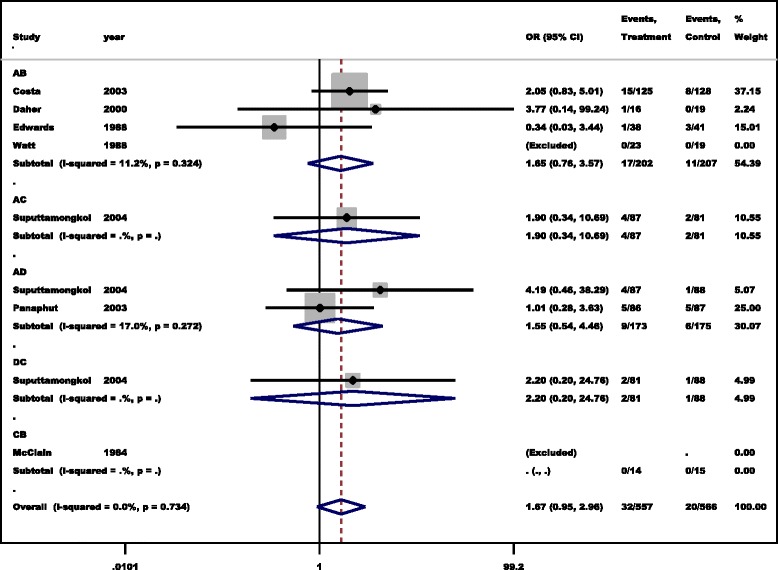
Forest plot showing the efficacy of antibiotics for the treatment of leptospirosis in a pairwise meta- analysis

In the direct comparison using a pairwise meta-analysis showed that there were comparable efficacies of antibiotics for the treatment of leptospirosis based on the mortality outcome. With regard to head-to head comparison, four studies [22–25] provided data on mortality in the penicillin group (17/202, 84.16%) and the placebo group (11/207,53%); a pooled analysis showed a comparable efficacy on mortality outcome between penicillin and placebo (OOR : 1.65, 95% CI: 0.76-3.52, *I* ^2^:11.2%)., Two studies [2, 26] reported data on mortality in the cephalosporin group (9/173, 52%) and the penicillin group (6/175,34.3%) and a pooled analysis showed a comparable efficacy on mortality outcome between these two drugs (OR: 1.55, 95% CI: 0.54-4.48, *I* ^*2*^: 17%). One each study compared penicillin and doxycycline (4/87 vs 2/81, OR: 1.9, 95% CI: 0.34-10.69) [2], doxycycline and cephalosporin (2/81 vs 1/88, OR: 2.2, 95% CI: 0.34-10.69) [26] or doxycycline and placebo (0/14 vs 0/15) [27], showing no differences in mortality outcome among the drugs of interest (Fig. 3). Overall, the absence of heterogeneity reflects the small number of included studies for pairwise comparison.

### The leptospirosis network

**Fig. 4:**
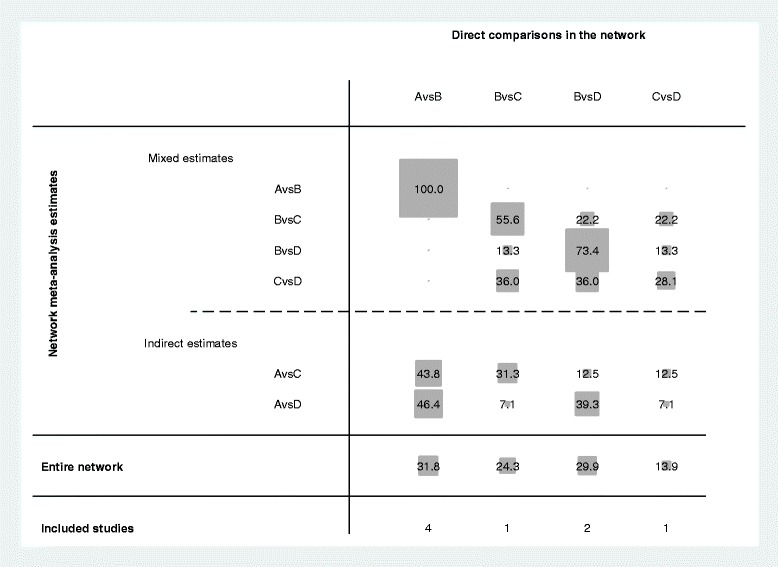
Contribution plot for the efficacy of antibiotics in treating leptospirosis

The input data for the current NMA is shown in Table 2. Figure 4 shows the contribution of each direct comparison in the network estimates. The comparison of penicillin versus the placebo (A vs B) has the largest contribution to the entire network (31.8%).

**Table 2:** The matrix of source data used in a network meta-analysis of antibiotic treatment of leptospirosis

Study, year [ref]	dA	nA	dB	nB	dC	nC	dD	nD
Suputtamongkol, 2004 [2]	4	87			2	81	1	88
Edwards,1988 [22]	1	38	3	41				
Watt,1988 [23]	05	23	0	19				
Daher, 2000 [24]	1	16	0	19				
Costa, 2003 [25]	15	125	8	128				
Panaphut, 2003 [26]	5	86					5	87
McClain,1984 [27]			0	15	0	14		

*d* number of deaths, *n* total number of patients with leptospirosis, *A* penicillin, *B* placebo, *C* doxycycline, *D*cephalosporin

**Fig. 5:**
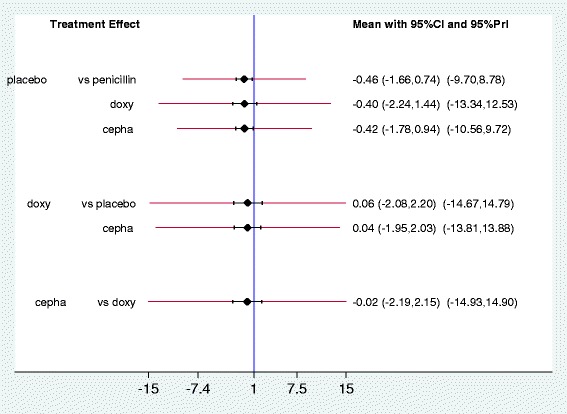
Predictive intervals plot for the antibiotic network from seven randomised controlled trials of the treatment of leptospirosis

A multivariate meta-analysis showed that there was no evidence of inconstency (*Chi* ^2^: 1.11;. Prob > *Chi* ^2^ : 0.29). *Tau* ^2^ values also showed an ‘agreement’ between the direct and indirect evidence (0.0031). The predictive interval plot (Fig. 5) indicates that for these comparisons (penicillin vs placebo, cephalosporin vs placebo) are wide enough compared with the CIs; this suggests that in a future study the active treatment can appear more effective than placebo.

**Fig. 6:**
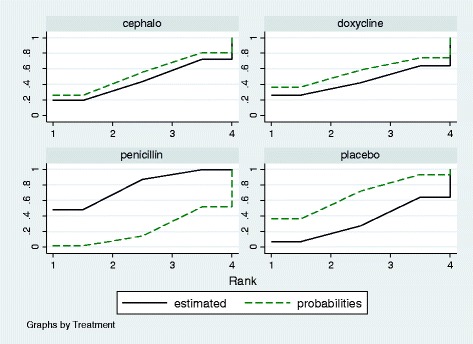
Plots of the surface under the cumulative ranking curves for all treatments in leptospirosis

The assessment of rank probabilities using SUCRA plots indicated that penicillin presented the greatest likelihood of improving efficacy, among the evaluated antibiotics for treating leptospirosis (Fig. 6).

## Discussion

Meta-analyses comparing multiple treatments are feasible and should be considered as the bedrock for decisions when several treatments are available [2, 4]. NMA in its standard form makes an assumption of ‘consistency’ [19] that estimates of treatment effects from direct and indirect evidence are in agreement [17, 19]. The current NMA could hold the key assumption of consistency.

The results of this NMA showed that it is possible to assess the efficacy of cephalosporin compared to a placebo even though this direct comparison was not performed in any of the included trials. Our results predicted that a cephalosporin antibiotic would have comparable efficacy to penicillin in reducing mortality in human leptospirosis. Indications for the use of cephalosporin antibiotics for the treatment of leptospirosis are included in the WHO guideline for management of leptospirosis [28] as well as some national guidelines for management of leptospirosis in some countries such as Malaysia, as an example [29].

A Cochrane review on seven RCTs [30] as well as a non-Cochrane review on ten RCTs [31] performed pairwise analyses of the efficacy of antibiotics for the treatment of human leptospirosis. Both reviews reported comparable efficacy of antibiotics in preventing mortality as an outcome as well as an effect on the duration of illness. The Cochrane systematic review concluded that there were insufficient evidence to advocate for or against the use of antibiotics for the treatment of treating leptospirosis [30] and the review by Charan and associates [31] showed that there was no significant difference between mortality in groups given penicillin compared to control groups.

The WHO treatment guidelines still recommend administration of antibiotics for leptospirosis regardless of the stage or severity of the disease [28]. The optimal treatment of leptospirosis remains a major clinical dilemma, for which limited data from clinical studies exist [14]. Penicillin G sodium (penicillin G) is generally recommended as the first choice treatment for severe leptospirosis. It is important to evaluate alternatives to penicillin G because its use has potential drawbacks. Antibiotic resistance has compromised the efficacy of penicillin G against many important bacterial pathogens, and it is intrinsically inactive against coinfected *Rickettsiosis* that are common in tropical areas such as Thailand [26]. In addition, Jarisch-Herxheimer Reaction (JHR) is a known complication associated with the use of penicillin G for the treatment of leptospirosis [32, 33]. Therefore, penicillin G administration might pose a great burden in critically ill patients [33]. Of note is that the small number of included studies and these being not recent is a reflection of the limited scientific interest in performing clinical trials in this field. There may be a number of reasons for this. For instance, the lack of a widely available, sensitive and rapid method of laboratory confirmation of leptospirosis has been an important impediment [2] and this compromises the recruitment of patients for the clinical trials. Moreover, there may be a concern whether the clinical manifestation of leptospirosis would become worse after the initiation of antibiotic therapy due to the development of JHR. A systematic review of 27 studies in JHR had reported the development of JHR in 92 of 976 leptospirosis patients within 1 to 48 h after administration of the first dose of antibiotic [32]. It is also noted that a higher proportion of JHR occurred in early stage leptospirosis, suggesting a higher probability of the (adverse) event before the natural clearance of spirochetes [32].

Other classes of antibiotic may provide better alternatives to penicillin G. Doxycycline has the advantage that it can be administered orally but it is not suitable in pregnant women. Like penicillin, most cephalosporin act on the bacterial cell wall synthesis, with some exceptions that act on protein synthesis [34]. Ceftriaxone can be administered once daily, which is an advantage over another third generation cephalosporin such as cefotaxime [2] and no dosage adjustment, was required for renal failure. In addition, there is no reported evidence of JHR in patients with leptospirosis. Moreover, ceftriaxone can give extra benefit of being an excellent empirical therapy for other infections (e.g. *Streptococcus pneumonia*) which mimic the clinical presentation of leptospirosis [14].

This is the first time that an indirect evaluation of the efficacy of an antibiotic treatment for leptospirosis using NMA has been performed. This is an important additional work because the evaluation of antibiotic treatments for leptospirosis using double-blind RCTs is complicated by ethical considerations associated with the provision of a placebo to severely affected patients [26]. Therefore, study designs that permit the use of indirect analyses of efficacy such as the NMA would allow an assessment of cephalosporin (ceftriaxone in this case) compared to a placebo control.

The indirect comparisons in the current review revealed that the antibiotics did not differ from each other with regards to their ability to reduce mortality, supporting the findings of earlier reviews [30, 31]. However, the NMA provided slightly different results compared to the more simplistic direct comparison using conventional pairwise meta-analysis efficacy estimates. This shows the potential advantage of NMA because it can incorporate both direct and indirect comparisons, decreasing the risk for possible sponsorship bias [35], which often is an issue for drug trails.

There are some limitations that needed to acknowledge. We did not find evidence of inconsistency in the results from our indirect comparison analysis. However, these findings should be interpreted with caution as only a small number of trials could be identified for inclusion in the current analysis. Nevertheless, our findings agree with the earlier reviews, indicating no significant difference between the antibiotics for mortality as an end point. The current network meta-analysis could hold the key assumption of consistency. The indirect comparisons presented in this study add to the current body of evidence in literature.

## Conclusions

Findings suggest that network meta-analysis, a meta-analysis comparing multiple treatments, is feasible and should be considered as better precision of effect estimates for decisions when several antibiotic options are available for the treatment of leptospirosis.

## Additional files

Additional file 1: Table S1. Citations and Ovid MEDLINE (R) <1946 to Present>. (DOC 47 kb)
Additional file 2: Table Characteristics of the clinical trials included in meta-analysis. (DOC 39 kb)

## Abbreviations

CENTRAL: Cochrane Central Register of Controlled Trials
JHR: Jarisch-Herxheimer reaction
NMA: Network meta-analysis
RCT: Randomized controlled trial
RoB: Risk of bias
SUCRA: Surface under the cumulative ranking curve

## References

[1] Liberati A, Altman DG, Tetzlaff J, Mulrow C, Gøtzsche PC, Ioannidis JPA (2009). The PRISMA statement for reporting systematic reviews and meta-analyses of studies that evaluate healthcare interventions: explanation and elaboration. BMJ.

[2] Suputtamongkol Y, Niwattayakul K, Suttinont C, Losuwanaluk K, Limpaiboon R, Chierakul W (2004). An open, randomized, controlled trial of penicillin, doxycycline, and cefotaxime for patients with severe leptospirosis. Clin Infect Dis.

[3] Caldwell DM, Ades AE, Higgins JP (2005). Simultaneous comparison of multiple treatments: combining direct and indirect evidence. BMJ.

[4] Smith CT, Marson AG, Chadwick DW, Williamson PR (2007). Multiple treatment comparisons in epilepsy monotherapy trials. Trials.

[5] Higgins JP, Whitehead A (1996). Borrowing strength from external trials in a meta-analysis. Stat Med.

[6] Glenny AM, Altman DG, Song F, Sakarovitch C, Deeks JJ, D’Amico R (2005). Indirect comparisons of competing interventions. Health Technol Assess.

[7] Li T, Puhan MA, Vedula SS, Singh S, Dickersin K (2011). Network meta-analysis-highly attractive but more methodological research is needed. BMC Med.

[8] Kirkham JJ, Riley RD, Williamson PR (2012). A multivariate meta-analysis approach for reducing the impact of outcome reporting bias in systematic reviews. Stat Med.

[9] Torgerson PR, Hagan JE, Costa F, Calcagno J, Kane M, Martinez-Silveira MS (2015). Global burden of leptospirosis: estimated in terms of disability adjusted life years. Plos Negl Trop Dis.

[10] Goris MGA, Kikken V, Straetemans M, Alba S, Goeijenbier M, van Gorp ECM (2013). Towards the burden of human leptospirosis: duration of acute illness and occurrence of post-leptospirosis symptoms of patients in the Netherlands. Plos One.

[11] Lozano R, Naghavi M, Foreman K, Lim S, Shibuya K, Aboyans V (2012). Global and regional mortality from 235 causes of death for 20 age groups in 1990 and 2010: a systematic analysis for the global burden of disease study 2010. Lancet.

[12] Costa F, Hagan JE, Calcagno J, Kane M, Torgerson P, Martinez-Silveira MS (2015). Global morbidity and mortality of leptospirosis: a systematic review. PLoS Negl Trop Dis.

[13] Levett PN (2001). Leptospirosis. Clin Microbiol Rev.

[14] Raptis L, Pappas G, Akritidis N (2006). Use of ceftriaxone in patients with severe leptospirosis. Int J Antimicrob Agents.

[15] Higgins JPT, Green S, editors. Cochrane Handbook for Systematic Reviews of Interventions Version 5.1.0 [updated March 2011]. The Cochrane Collaboration, 2011. Available from: http://handbook.cochrane.org.

[16] Chiodo GT, Tolle SW, Bevan L (2000). Placebo-controlled trials: good science or medical neglect?. West J Med.

[17] Jansen JP, Fleurence R, Devine B, Itzler R, Barrett A, Hawkins N (2011). Interpreting indirect treatment comparisons and network meta-analysis for health-care decision making: report of the ISPOR task force on indirect treatment comparisons good research practices: part 1. Value Health.

[18] Cope S, Zhang J, Saletan S, Smiechowski B, Jansen JP, Schmid P (2014). A process for assessing the feasibility of a network meta-analysis: a case study of everolimusin combination with hormonal therapy versus chemotherapy for advanced breast cancer. BMC Med.

[19] White IR, Barrett JK, Jackson D, Higgins JPT (2012). Consistency and inconsistency in network meta-analysis: model estimation using multivariate meta-regression. Res Synth Methods.

[20] Chaimani A, Higgins JPT, Mavridis D, Spyridonos P, Salanti G (2013). Graphical tools for network meta-analysis in STATA. PLoS One.

[21] White IR (2011). Multivariate random-effects meta-regression: updates to *mvmeta*. STATA J.

[22] Edwards CN, Nicholson GD, Hassell TA, Everard CO, Callender J (1988). Penicillin therapy in icteric leptospirosis. Am J Trop Med Hyg.

[23] Watt G, Padre LP, Tuazon ML, Calubaquib C, Santiago E (1988). Placebo-controlled trial of intravenous penicillin for severe and late leptospirosis. Lancet.

[24] Daher EF, Nogueira CB (2000). Evaluation of penicillin therapy in patients with leptospirosis and acute renal failure. Rev Inst Med Trop Sao Paulo.

[25] Costa E, Lopes AA, Sacramento E, Costa YA, Matos ED, Lopes MB (2003). Penicillin at the late stage of leptospirosis: A randomized controlled trial. Rev Inst Med Trop Sao Paulo.

[26] Panaphut T, Domrongkitchaiporn S, Vibhagool A, Thinkamrop B, Susaengrat W (2003). Ceftriaxone compared with sodium penicillin g for treatment of severe leptospirosis. Clin Infect Dis.

[27] McClain JB, Ballou WR, Harrison SM, Steinweg DL (1984). Doxycycline therapy for leptospirosis. Ann Intern Med.

[28] World Health Organization (2003). Human leptospirosis: guidance for diagnosis, surveillance and control.

[29] The Malaysian, Ministry of Health (2011). Guidelines for the diagnosis, management, prevention and control of leptospirosis in Malaysia.

[30] Guidugli F, Castro AA, Atallah AN (2000). Antibiotics for treating leptospirosis. Cochrane Database Syst Rev.

[31] Charan J, Saxena D, Mulla S, Yadav P (2013). Antibiotics for the treatment of leptospirosis: systematic review and meta-analysis of controlled trials. Int J Prev Med.

[32] Guerrier G, D’Ortenzio E (2013). The jarisch-herxheimer reaction in leptospirosis: a systematic review. Plos One.

[33] Emmanouilides CE, Kohn OF, Garibaldi R (1994). Leptospirosis complicated by a Jarisch-Herxheimer reaction and adult respiratory distress syndrome: case report. Clin Infect Dis.

[34] Ghooi RB, Thatte SM (1995). Inhibition of cell wall synthesis--is this the mechanism of action of penicillins?. Med Hypotheses.

[35] Cipriani A, Santilli C, Furukawa TA, Signoretti A, Nakagawa A, McGuire H, Churchill R, Barbui C (2009). Escitalopram versus other antidepressive agents for depression. Cochrane Database Syst Rev.

