# Supplementary material for: Comparing antibiotic treatment for leptospirosis using network meta-analysis: a tutorial
Source: BMC Infect Dis. 2017 Jan 5;17:29. doi: 10.1186/s12879-016-2145-3 (PMC5217240; doi:10.1186/s12879-016-2145-3)
Supplement: Additional file 1: Table S1. — Citations and Ovid MEDLINE (R) <1946 to Present>. (DOC 47 kb) [file 12879_2016_2145_MOESM1_ESM.doc]

**Additional file 1: Table S1**. Citations and Ovid MEDLINE(R) <1946 to Present>

| Search terms**:** 1 | "randomized controlled trial".pt. (513377) |
| --- | --- |
| 2 | (random$ or placebo$ or single blind$ or double blind$ or triple blind$).ti,ab. |
| 3 | (retraction of publication or retracted publication).pt. |
| 4 | 1 or 2 or 3 |
| 5 | (animals not humans).sh. |
| 6 | ((comment or editorial or meta-analysis or practice-guideline or review or letter or journal correspondence) not "randomized controlled trial").pt. |
| 7 | (random sampl$ or random digit$ or random effect$ or random survey or random regression).ti,ab. not "randomized controlled trial".pt. |
| 8 | 5 or 6 or 7 |
| 9 | 4 not 8 |
| 10 | (random$ or placebo$ or single blind$ or double blind$ or triple blind$).ti,ab. |
| 11 | RETRACTED ARTICLE/ |
| 12 | 10 or 11 |
| 13 | (animal$ not human$).sh,hw. |
| 14 | (book or conference paper or editorial or letter or review).pt. not exp randomized controlled trial/ |
| 15 | (random sampl$ or random digit$ or random effect$ or random survey or random regression).ti,ab. not exp randomized controlled trial/ |
| 16 | 13 or 14 or 15 |
| 17 | 12 not 16 |
| 18 | 9 or 17 |
| 19 | exp leptospirosis/ |
| 20 | ((lepto$)).tw. |
| 21 | exp leptospiral infection/ |
| 22 | 19 or 20 or 21 |
| 23 | (sever$ or terminal or uncomplicate or nonseve).ti,ab |
| 24 | 22 and 23 |
| 25 | Antibiotic.mp |
| 26 | exp Drug Therapy/ |
| 27 | (antibiotic and therap*).mp. [mp=ti, ab, sh, hw, tn, ot, dm, mf, dv, kw, nm, kf, ps, rs, ui] |
| 28 | (penici* or doxycy*or cephalo* or ampi*or chloro* or arzithr* |
| 29 | 25 or 26 or 27 or 28 |
| 30 | 18 and 24 and 29 |
| 31 | limit 30 to english language |
| 32 | limit 31 to human |
| 33 | limit 32 to humans |
| 34 | remove duplicates from 33 |
