# Supplementary material for: Comparing antibiotic treatment for leptospirosis using network meta-analysis: a tutorial
Source: BMC Infect Dis. 2017 Jan 5;17:29. doi: 10.1186/s12879-016-2145-3 (PMC5217240; doi:10.1186/s12879-016-2145-3)
Supplement: Additional file 2: — Table Characteristics of the clinical trials included in meta-analysis. (DOC 39 kb) [file 12879_2016_2145_MOESM2_ESM.doc]

Additional file 2 Table Characteristics of the clinical trials included in meta-analysis

| Study,  year | Study period | Country | Participant | Male% | Diagnostic test | Age in year | Severity | Day of treatment initiation | Experimental drug | Comparator | Dosage | Route | Outcome^2^ | Follow-up |
| --- | --- | --- | --- | --- | --- | --- | --- | --- | --- | --- | --- | --- | --- | --- |
| Suputtamongkol 2004 [2] | 7/ 2001 -12/2002 | Thailand | 264 | 89.5% | NA | NA | severe | on admission | Penicillin | Doxy vs Cefotaxime | .5 MU 6hrly vs 1G,  6hrly  vs 200 mg infused 30 min ^1^ | IV | mortality |  |
| Edwards,1988 [22] | 10/1983-12/1986 | Barbados | 79 |  | MAT; ELISA; urine culture |  | severe^3^ |  | Penicillin | Placebo (saline) | 6hrly x 5 d | IV | mortality, renal failure |  |
| Watt, 1988 [23] | 9-11/1985 & 7-10/ 1986 | Philippines | 42 | 87% | DOT-ELIZAELISA | 8.6  (19-52) | severe | before admission, | Penicillin G |  | 6 MU/d x 7d | IV | zero mortality; no dialysis; fever resolution | 1 wk, 1 mth |
| Dahler 2000 [24] | 5/1996- to 6/1998 | Brazil | 35 | 86% | IgM | 35± 10 | severe / ARF | on admission | Penicillin | no antibiotics | 6 MU/d x 8d | IV | mortality | 4d |
| Costa, 2003 [25] | 8.1997-7/1999 | Brazil | 253 | 88% | MAT | 35.8± 13.9 | severe | NA | Penicillin | placebo | 6MU,  4hrly x 7d | IV | mortality |  |
| Panaphut 2003 [26] | 7/ 2000-  12/ 2001 | Thailand | 87 | NA | IgM | 42 (31-53) | severe | on admission | Ceftriaxone | Penicillin G | 1 g daily x 7 d vs  1.5 MU 6hrly x 7d. | IV vs IV | mortality ; fever resolution;  renal failure | NA |
| McClain,1984 [27] | NA | USA | 29 | NA | MAT | NA | non-severe | on admission | Doxy | Placebo | twice daily x 7d | oral | zero mortality; fever resolution |  |

^1^Followed by gentamycin 100 mg; ^2^: more significant outcomes; 3: with jaundice/ renal failure; ARF: acute renal failure; DOT-ELIZA: Dot-enzyme linked immunosorbent assay; Doxy; doxycycline; MAT: microaggultination test; MU: million units; NA: not available/ not reported
